# US Presidential Party switches are mirrored in global maternal mortality

**DOI:** 10.1136/bmjgh-2025-020223

**Published:** 2026-03-24

**Authors:** Sonia Bhalotra, Damian Clarke, Manuel Fernandez Sierra, Hanna Mühlrad

**Affiliations:** 1 Department of Economics, University of Warwick, Coventry, UK; 2 Department of Economics, University of Exeter, Exeter, UK; 3 Department of Economics, Universidad de Chile, Santiago, Chile; 4 Department of Economics, Universidad de los Andes, Bogotá, Colombia; 5 Department of Global Public Health, Karolinska Institutet, Stockholm, Sweden

**Keywords:** Maternal health, Health policy, Global Health

## Abstract

In this observational study, we provide estimates of the impact on the maternal mortality ratio of swings in US aid for family planning and reproductive health driven by changes in the implementation of the Mexico City Policy—first introduced as the US Policy on Population Assistance under the Reagan administration in 1984 and often referred to as the Global Gag Rule (GGR). The policy prohibits US foreign assistance for family planning to overseas non-governmental organisations that provide, make referrals to or promote abortion-related services or information, even when financed through non-US funds. Since first implemented by President Reagan in 1984, the GGR has been enacted under every Republican president and revoked under every Democrat. The policy was tightened under President Trump in his first presidency in 2017 and expanded to apply to all US global health assistance. Using data for the period 1985–2023, covering 150 countries worldwide and using a quasi-experimental study design, we estimate that a switch from a Democratic to a Republican administration, for a country with above median reliance on US family planning aid, is associated with an additional 44.7 maternal deaths per 100 000 live births—an increase of 10.5%. This erodes roughly one-fifth of the average worldwide decline in maternal mortality achieved since 1985. Additionally, our findings offer suggestive evidence of potential mechanisms including a reduction in contraceptive use, an increase in unmet need for contraception and a decrease in the proportion of births attended by skilled health workers.

**WHAT IS ALREADY KNOWN ON THIS TOPIC**

- Access to abortion care is central to women’s health and wellbeing, and the GGR has interrupted a range of family planning programmes with follow-on impacts on service provision, fertility rates and access to health inputs.

**WHAT THIS STUDY ADDS**

- US family planning aid drops under Republican Presidents when the Global Gag Rule (GGR) is in force. Once Democratic Presidents are elected, it increases by 48%.
- Countries heavily reliant on US aid see a 10.5% increase in maternal mortality following a switch from a Democratic to a Republican administration—about 44.7 additional deaths per 100 000 live births. This mortality increase erodes one-fifth of the decline in global maternal mortality decline achieved since 1985.
- A US$1 per capita higher reliance on US Overseas Development Assistance for reproductive and maternal health relative to a mean level of US$0.872 per capita is associated with 25 additional maternal deaths per 100 000 live births under the GGR.
- An increase in the maternal mortality ratio is seen in each of the country regions. Although Africa experiences the largest impact in absolute terms, the percentage impact is greatest in Latin America (16%), followed by Asia (15%) and Africa (7%).

**HOW THIS STUDY MIGHT AFFECT RESEARCH, PRACTICE OR POLICY**

- These findings highlight the importance of foreign aid for maternal health outcomes and highlight how abrupt policy shifts in donor countries can substantially undermine progress in maternal mortality reduction

## Background

 Under the Helms Amendment (1973), US law prohibits the use of foreign aid to perform induced abortions as a method of family planning or to motivate or coerce any person to undergo an abortion. In 1984, at an international convention in Mexico City, President Ronald Reagan introduced the US Policy on Population Assistance (the Mexico City Policy), which restricted US global family planning assistance to overseas non-governmental organisations (NGOs) that provide information, counselling or referrals regarding induced abortion, or that advocate for abortion rights. Because of its effect on limiting the ability of reproductive health organisations to discuss or support abortion-related services, the policy is commonly referred to as the Global Gag Rule (GGR), as it effectively ‘gags’ organisations from engaging in abortion-related health and rights activities.^1^,^3^ Ever since the policy was first implemented by President Reagan, it has been reinstated under every Republican administration and revoked under every Democratic administration.^1^

US aid policy has a substantial impact on total global health assistance. For instance, in 2022 the USA provided 51% of all Overseas Development Assistance (ODA) for health from Development Assistance Committee members, with bilateral disbursements amounting to approximately US$ 13.4 billion between 2021-2022.^4^ ^5^ The USA also plays a critical role in ODA directed towards family planning and reproductive health. Consequently, reproductive healthcare and rights in developing countries are closely tied to shifts in US Presidential politics.^6^

Under the first Trump administration (2017–2020), the GGR—rebranded as the Protecting Life in Global Health Assistance policy^7 8^—was not only reinstated but also significantly expanded to apply to all US global health assistance. This expansion extended the restrictions to foreign NGOs that used their own non-US funds to provide, counsel, refer or advocate for abortion-related services.^1^ The second Trump administration has initiated sweeping changes to US global health assistance in 2025, including substantial cuts to foreign aid and a major restructuring of the United States Agency for International Development (USAID). Beginning in early 2025, the administration paused many USAID programmes, terminated a large share of awards and announced plans to merge remaining functions into the State Department. It is estimated that over 90% of all USAID awards for family planning and reproductive health programmes have been terminated.^9^ As a result, access to reproductive health services in developing countries now faces more severe challenges than ever before, particularly in the provision of family planning and related care.^10^

Beyond abortion-related services, the policy produces negative spillover effects for organisations working in other health domains due to misunderstandings about the requirements of the rule. Fearing the loss of US funding, many organisations have experienced a ‘chilling effect’, restricting activities beyond what the GGR actually mandates.^2 11 12^ Thus, the GGR’s reach extends into other areas of public health, including HIV/AIDS prevention, where it has constrained service delivery. Moreover, evidence suggests that the policy’s impacts may persist even after its repeal due to stigma and institutional inertia.^12^

Much of the valuable existing work on the GGR consists of field studies conducted in a single country—for instance, demonstrating clinic closure. We build on the smaller body of quantitative research using large representative samples. Existing studies of the GGR show that the policy has failed to achieve its main objective—namely, reducing induced abortion rates.^2 13 14^ Instead, it has disrupted funding for family planning programmes leading to clinic closures, staff layoffs, reduced access to family planning services^15 16^ and higher rates of unmet contraceptive need.^17^ Reduced access to contraception and to family planning advice have together contributed to an increase in unintended pregnancies,^15^ induced abortions,^2 13 14^ HIV infection rates and infant mortality.^18^ The first estimates for maternal mortality were provided by Bhalotra *et al.*^19^ and subsequently by Kavakli *et al*.^18^ In this paper we extend the data forward by a decade to 2024 and provide pooled estimates and also regional estimates for Africa, Asia and Latin America.

Maternal mortality is an indicator of a nation’s overall health, the quality and accessibility of its healthcare system and the status of women in society.^20^ It is closely linked to unsafe abortion and reduced access to essential health services. An increased demand for abortion flowing from reduced access to contraception at a time when already limited reproductive health services are being scaled back is likely to have resulted in a rise in unsafe procedures. Although unsafe abortion is difficult to measure accurately due to its often clandestine nature, it is estimated to account for between 4.7% and 13.2% of maternal deaths worldwide.^21^ It is therefore plausible that increases in unsafe abortion will manifest as higher maternal mortality rates. In addition, the total impact of the GGR on maternal mortality is likely amplified by its broader effects on other areas of health service provision including antenatal care and HIV prevention. The causal mechanisms underlying our analysis are conceptually summarised in online supplemental figure A.1.

## Methods

### Data on maternal health and reproductive outcomes

We constructed a country–year panel using data from the World Bank’s World Development Indicators (WDI) for all years between 1985 and 2023 in which the relevant variables are reported. Our primary outcome of interest is maternal mortality. The maternal mortality ratio (MMR), (variable code SH.STA.MMRT), is defined as the number of women who die from pregnancy-related causes while pregnant or within 42 days of pregnancy termination per 100 000 live births.^22^ These data are estimated using Bayesian modelling that incorporates information on the proportion of maternal deaths among non-AIDS-related deaths in women of reproductive age (15–49 years), fertility, the share of births attended by skilled health personnel and GDP measured using purchasing power parities. The estimates are produced following the methodology developed by the Maternal Mortality Estimation Inter-Agency Group, which comprises the World Health Organization, the United Nations Children’s Fund, the World Bank and the United Nations Population Fund.

Our secondary outcomes of interest, which capture intermediate effects of the GGR, include modern contraceptive prevalence, unmet need for contraception and the percentage of births attended by skilled health personnel. These data are also retrieved from the World Bank Group’s WDI. Modern contraceptive prevalence (variable code SP.DYN.CONM.ZS) is defined as the percentage of married women of reproductive age (15–49 years) who are using, or whose partners are using, any modern contraceptive method, including female and male sterilisation, oral hormonal pills, intrauterine devices, male condoms, injectables, implants (including Norplant), vaginal barrier methods, female condoms and emergency contraception.^23^ Unmet need for contraception (variable code SP.UWT.TFRT) is defined as the percentage of fertile married women of reproductive age (15–49 years) who do not want to become pregnant and are not using contraception.^24^ Data on unmet need for contraception are available only for a subset of our sample, covering 57 countries. Births attended by skilled health personnel (variable code SH.STA.BRTC.Z) is defined as the percentage of deliveries attended by staff trained to provide the necessary supervision, care and advice to women during pregnancy, childbirth and the postpartum period; to conduct deliveries independently; and to care for newborn infants.^25^

In online supplemental table A.4 we show data availability by country, exposure classification and the number of non-missing observations for each outcome.

### Maternal health aid and exposure to the Global Gag Rule (GGR)

The outcome data are merged with data on each country’s aid receipts for health, including the channel to which the aid is directed (including maternal health or the sub-channel of maternal health specifically directed to family planning), as well as the source of these receipts (Institute for Health Metrics and Evaluation, 2018).^26^ All receipts are expressed in current US dollars in 2019, thus accounting for inflation. The GGR was switched on in Republican presidential periods starting with the original policy announcement by Reagan in 1984 and switched off (repealed) in each Democratic presidency, generally on the President’s first full day in office in late January. Thus, the GGR is switched on during Republican presidential regimes after the 1984 announcement (1985–1992; 2001–2008; 2017–2020) and switched off during Democratic presidential regimes (1993–2000; 2009–2016; 2021–2024). The analysis sample is restricted to countries that receive some reproductive or maternal health aid from US official development assistance in at least 1 year between 1985 and 2019.

We measure a country’s exposure to the GGR in two ways. First, following *Bendavid et al*,^13^ we construct a binary indicator (${High}_{c}$) that equals 1 for countries whose per-capita receipts of US family planning aid during the Obama administration (2009–2016) are at or above the sample median; countries receiving less than the median amount serve as the low-dependence comparison group. Aid in the Obama period provides a natural proxy for potential losses under the GGR because aid coverage is most complete in these years. However, in robustness checks we show that our results are similar when exposure is defined using aid received during the Clinton administration. A map of exposure to the GGR under the Obama presidency is shown in online supplemental figure A.2. The map depicts countries classified as highly exposed (in red) and those classified as having low exposure to the policy (in blue). Second, we construct a continuous ‘dose–response’ measure of exposure, ${Aid}_{c}$, defined as reproductive and maternal health aid per capita during the Obama period, which exploits variation in the magnitude of aid receipts across countries.

Table 1 shows that countries in our sample differ sharply in both their exposure to US reproductive and maternal health aid and their baseline maternal health outcomes. On average, countries received about US$0.87 in reproductive and maternal health aid per capita during the Obama period, but this masks large heterogeneity: high-exposure countries receive roughly US$1.66 per capita compared with only about US$0.10 in low-exposure countries.

**Table 1:** Maternal health outcomes and aid exposure: summary statistics

	Full sample	By aid exposure		Continents		
		Low	High	Africa	Latin America	Asia
Reproductive and maternal health aid, US$ (*Average per capita in Obama period 2019*)	0.872 (1.256)	0.099 (0.105)	1.656 (1.397)	1.103 (1.177)	0.615 (0.897)	0.769 (1.460)
Maternal mortality ratio (*per 100 000 live births*)	293.8 (438.3)	160.0 (256.0)	427.7 (531.9)	522.9 (565.7)	112.8 (112.9)	149.7 (244.9)
Births attended by skilled health personnel (*% of live births*)	85.7 (22.2)	92.9 (15.1)	75.3 (26.2)	65.6 (25.7)	92.1 (13.8)	90.6 (19.4)
Contraceptive prevalence, modern methods (*% women aged 15–49 years*)	36.1 (20.4)	43.6 (19.8)	29.8 (18.7)	25.5 (18.0)	52.1 (15.9)	41.3 (18.0)
Unmet need for contraception *(% of married women aged 15–49 years)*	11.6 (8.5)	10.7 (7.2)	11.8 (8.9)	9.3 (7.3)	22.3 (8.0)	10.0 (6.0)

Table shows sample means along with standard deviations (SD) in parentheses.Appendix Table A.4

Column (1) uses all available country–year observations. Columns (2) and (3) restrict the sample to low- and high-exposure countries, respectively, while columns (4), (5) and (6) restrict the sample by continent.

High-exposure countries are defined as those with reproductive and maternal health aid per capita above the sample median during the Obama period (2009–2016).

The number of observations may differ across rows because of missing data in the underlying WDI indicators (see online supplemental appendix table A.4).

The analysis is restricted to countries that received some reproductive or maternal health aid from US official development assistance in at least 1 year between 1985 and 2019.

Consistent with aid being targeted towards worse-off settings, high-exposure countries have substantially poorer maternal health indicators. Their average MMR is 428 deaths per 100 000 live births, 2.6 times larger than the 160 deaths per 100 000 observed in low-exposure countries. High-exposure countries also have a lower modern contraceptive prevalence (about 30% vs 44% of women aged 15–49 years), lower coverage of skilled birth attendance (75% vs 93% of live births) and a higher unmet need for contraception (11.8 vs 10.7 births per woman). Patterns by continent echo these gradients: African countries receive the largest amounts of reproductive and maternal health aid per capita, they have higher maternal mortality and a relatively low coverage of skilled birth attendance and modern contraception while Latin American countries, at the other end of the spectrum, have relatively low maternal mortality with high skilled birth attendance and contraceptive prevalence. However, possibly related to religious restrictions, they have a comparatively high unmet need for contraception. The relevant indicators for Asian countries lie between these two extremes. The descriptive data confirm that the countries most exposed to swings in US family planning aid are those starting out with markedly worse maternal health conditions.

### Research design and statistical analysis

The study uses a difference-in-differences design to estimate the causal impact of the GGR. In the binary exposure specification we compare changes in outcomes over time between countries that are highly exposed to the policy and those that are less exposed, where exposure is defined by their reliance on US family planning aid at the time the policy is not enacted (ie, during a Democratic regime). This empirical strategy follows prior work in the literature.^13 18 27 28^ For our primary outcome, MMR, we estimate:

.$$Maternal\;Mortality\;Rate_{ct}=\alpha +\beta \left(GGR_{t}\times High_{c}\right)+\delta_{c}+\lambda_{t}+\varepsilon_{ct}$$

Here the MMR from country c in year t is regressed on a measure of whether the GGR was ‘switched on’ in year t (${GGR}_{t}$) interacted with whether the country was highly exposed to the GGR (${High}_{c}$). Thus, β measures how much maternal mortality changes in high-exposure countries relative to low-exposure countries when the GGR is switched on, compared with periods when it is switched off. Country-specific time-invariant factors are captured in country-specific fixed effects ($\delta_{c}$), and secular changes in rates of maternal death are captured in year fixed effects $\lambda_{t}$. $\varepsilon_{ct}$ is a mean-zero unobserved stochastic error term. Our identifying assumption is that, in the absence of a change of Presidential administrations, areas which are highly exposed to the reform would have followed trends (though not levels) observed in areas which are less exposed to the reform.

The corresponding regression in the ‘dose-response’ specification is:


$$Maternal\;Mortality\;Rate_{ct}=\alpha +\beta \left(GGR_{t}\times Aid_{c}\right)+\delta_{c}+\lambda_{t}+\varepsilon_{ct}$$

where all definitions are as in the previous equation but now exposure is continuous, indicated by ${Aid}_{c}$.

For each secondary outcome we re-estimate the models (binary and continuous exposure) with MMR replaced by the relevant outcome variable: contraceptive use, proportion of births attended by a skilled health worker, and unmet need for contraception. We conduct several robustness checks to assess the stability of our estimates. First, we re-define baseline aid dependence using the Clinton administration period (1993–2000) rather than the Obama administration (2009–2016) to verify that the results are not sensitive to the choice of reference period. Second, we include additional time-varying covariates—GDP per capita and secondary school enrolment—to account for potential confounding trends in socioeconomic development. Third, we exclude the years 2020 and 2021, when the COVID-19 pandemic caused a substantial global increase in maternal mortality and disrupted health service delivery, to ensure that our results are not driven by the pandemic. These results are presented in online supplemental tables A1–3. Fourth, we note that, to the extent that other countries raised their foreign aid to developing countries to compensate for US aid cuts, our estimates capture the net impact of US aid cuts.

## Findings

**Figure 1:**
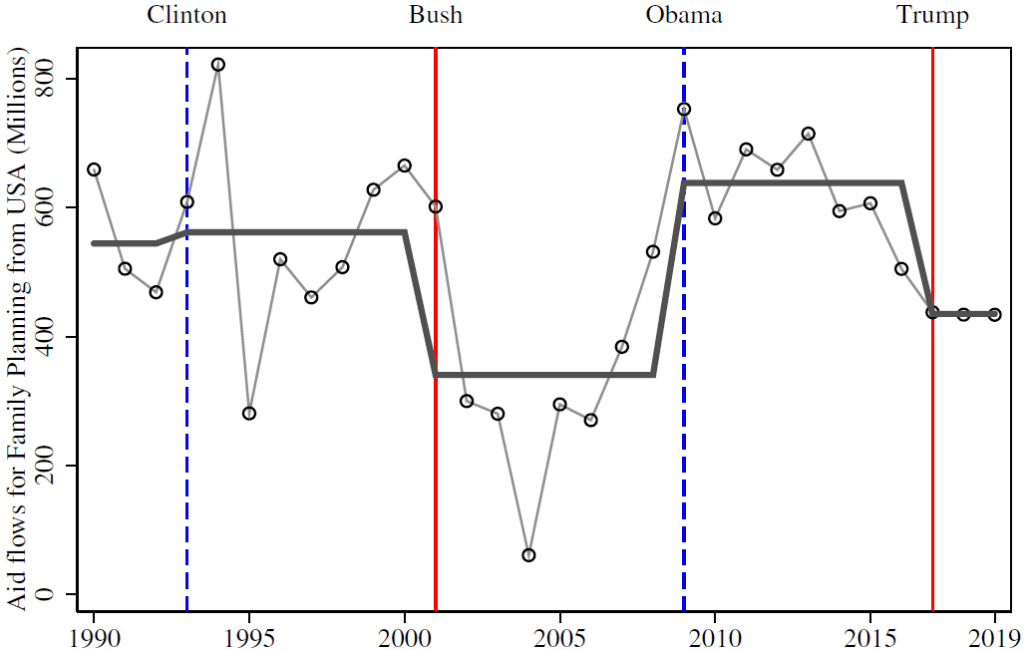
Total US aid flows for family planning. The figure shows total aid flows from the USA as Official Development Assistance (ODA) for health directed to family planning spending in 2019 US$ prices. The line connecting the circles shows yearly aid while the solid line shows President-specific averages. The Global Gag Rule (GGR) was switched on in all Republican presidential administrations and switched off in all Democratic administrations, with the exception of a short period during the Clinton administration. Vertical dashed lines mark years in which the US administration changes and the GGR is switched off (blue) or on (red).

We estimate that aid for family planning is, on average, 48% higher under Democratic than under Republican presidential administrations (figure 1). In per-capita terms, the swings are especially pronounced in high-exposure countries: among countries with above median baseline aid receipts, per-capita aid falls by about 34% in Republican Presidencies. For low-exposure countries the corresponding decline is only about 10%.

**Figure 2:**
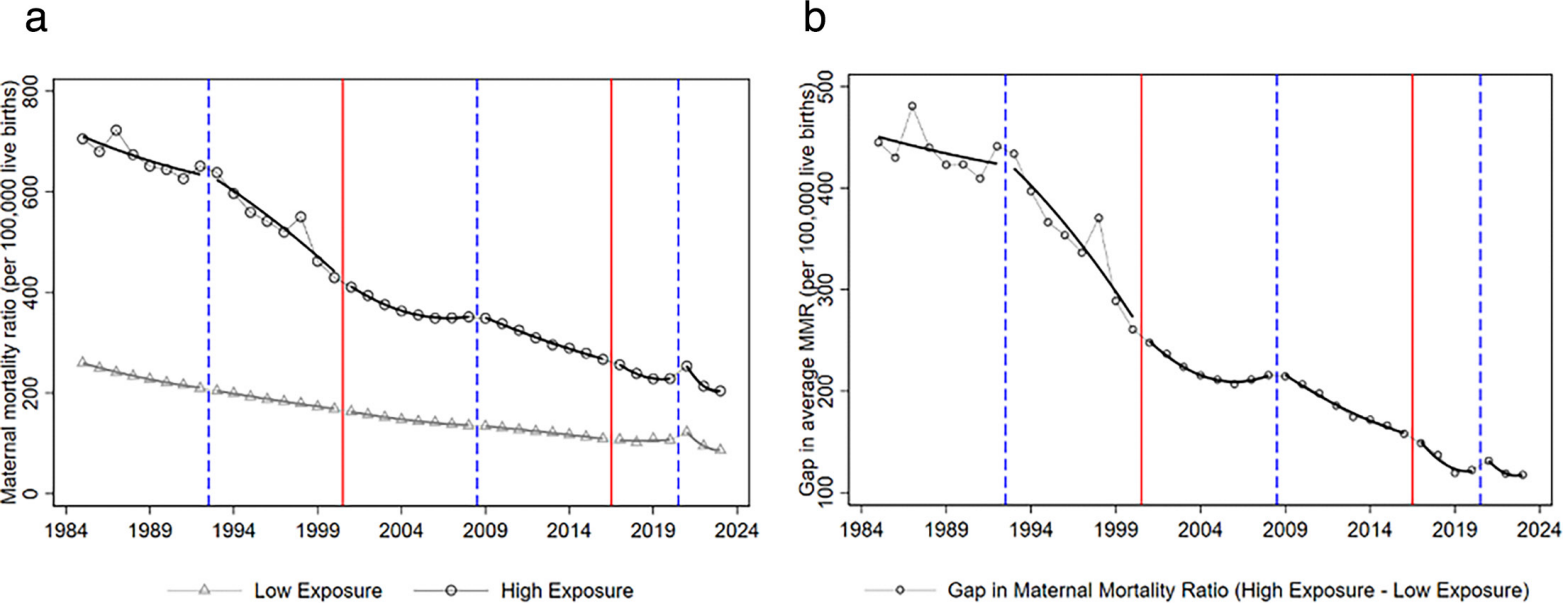
Maternal mortality over time by aid exposure, 1985–2023. The figure shows the evolution of the maternal mortality ratio (deaths per 100 000 live births) for countries with high and low exposure to US reproductive and maternal health aid. High-exposure countries are defined as those with reproductive and maternal health aid per capita above the sample median during the Obama period (2009–2016) and low-exposure countries are the remainder. (A) Yearly averages of the maternal mortality ratio for high- and low-exposure countries, together with a local polynomial fit of degree-2 for each series. (B) Yearly gap in average maternal mortality between high- and low-exposure countries (high minus low), again overlaid with a degree-2 local polynomial fit. Vertical dashed blue lines mark years in which the Global Gag Rule (GGR) is switched off (Democratic administrations) and vertical solid red lines mark years in which the GGR is switched on (Republican administrations).

Figure 2 shows that maternal mortality has fallen markedly in both high- and low-exposure countries over the past four decades. High-exposure countries start from substantially higher levels and remain worse off throughout, but they experience large declines. Low-exposure countries follow a lower trajectory overall, and the gap in average MMR between the two groups shrinks from roughly 445 to around 120 deaths per 100 000 live births. This underlying convergence of MMR across countries is consistent with there being more room for decline in countries with initially high levels and related to it being easier to bring MMR down when starting from a higher level (the ‘low hanging fruit’ argument).

Relevant to this study, figure 2 suggests that movements in maternal mortality are not uniform across political cycles. In panel (A) the decline in high-exposure countries shows noticeable changes in slope around presidential transitions marked by the vertical lines, whereas the low-exposure series follows a more steadily downward path. Panel (B) shows that the gap between high- and low-exposure countries narrows most visibly during periods when the GGR is not in force. These descriptive patterns motivate the difference-in-differences analysis that follows, which formally assesses whether GGR cycles are associated with systematic changes in maternal mortality.

A switch from a Democratic to a Republican administration—corresponding to the reinstatement of the GGR—is associated with a statistically significant rise in maternal deaths. Specifically, for countries with above median reliance on US family planning aid, a Republican presidency is linked to a 10.5% increase in maternal mortality compared with the mean of the dependent variable, equivalent to approximately 45 additional deaths per 100 000 live births (95% CI 20.38 to 69.18) (table 2A, column 1). The effect is consistently observed across regions including Africa, Latin America and Asia, indicating that the detrimental impact of the policy is not confined to a single geographical context (table 2A, columns 2–4). Although Africa experiences the largest impact in absolute terms, the percentage impact is greatest in Latin America (16%), followed by Asia (15%) and Africa (7%).

**Table 2:** Impact of the Global Gag Rule (GGR) on maternal mortality and reproductive health outcomes (1985–2023), binary exposure measure

	Full sample	Continents		
		Africa	Latin America	Asia
**(A) Maternal mortality ratio (per 100 000 live births)**				
High exposure×GGR	44.730***	43.829*	25.194**	39.264**
	(12.373)	(23.266)	(9.453)	(19.297)
	(20.281 to 69.180)	(−2.711 to 90.369)	(5.887 to 44.500)	(0.623 to 77.904)
Observations	5850	2379	1209	2262
Countries	150	61	31	58
Mean of Dep. Var. (high exposure)	427.7	626.9	154.4	269.8
Effect relative to mean Dep. Var. (%)	10.5	7.0	16.3	14.6
**(B) Births attended by skilled health personnel (% of live births)**				
High exposure×GGR	−0.676	0.242	−0.900	−0.732
	(0.604)	(1.720)	(0.871)	(1.091)
	(−1.870 to 0.518)	(−3.198 to 3.682)	(−2.680 to 0.879)	(−2.916 to 1.452)
Observations	2196	469	633	1089
Countries	150	61	31	58
Mean of Dep. Var. (high exposure)	75.3	59.5	86.3	79.6
Effect relative to mean Dep. Var. (%)	−0.9	0.4	−1.0	−0.9
**(C) Contraceptive prevalence, modern methods (% women aged 15–49 years)**				
High exposure×GGR	−1.182	−1.187	−2.200	−1.486
	(0.805)	(1.143)	(2.055)	(1.427)
	(−2.774 to 0.410)	(−3.473 to 1.100)	(−6.440 to 2.041)	(−4.352 to 1.379)
Observations	923	419	159	341
Countries	137	60	25	52
Mean of Dep. Var. (high exposure)	29.7	23.8	47.9	28.1
Effect relative to mean Dep. Var. (%)	−4.0	−5.0	−4.6	−5.3
**(D) Unmet need for contraception (% of married women age**d **15–49 years)**				
High exposure×GGR	1.608*	0.995	–	3.469**
	(0.864)	(1.459)	–	(1.075)
	(−0.123 to 3.340)	(−1.967 to 3.958)	–	(1.128 to 5.811)
Observations	226	139	–	45
Countries	57	36	–	13
Mean of Dep. Var.	11.7	9.4	–	8.9
Effect relative to mean Dep. Var. (%)	13.7	10.6	–	39.0

*p<0.10, **p<0.05, ***p<0.01.

Each column reports coefficients from regressions of maternal health outcomes on a country’s degree of exposure to the GGR interacted with whether the GGR is enacted.

High-exposure countries are defined as those with reproductive and maternal health aid per capita above the sample median during the Obama period (2009–2016).

Panel A uses the maternal mortality ratio (per 100 000 live births) as the dependent variable. Panel B uses the share of births attended by skilled health personnel (% of live births). Panel C uses contraceptive prevalence, modern methods (% of women aged 15–49 years). Panel D uses unmet need for contraception (% of married women aged 15–49 years).

Estimates for unmet need for contraception are not reported for Latin America due to insufficient data for that outcome in the region. Every regression includes country and year fixed effects (not shown) to capture country-level heterogeneity and common trends.

Robust standard errors clustered at the country level in parentheses with 95% CIs.

Dep. Var., dependent variable.

Using the continuous specification, measuring the dosage-response impact, a US$1 per capita higher reliance on US ODA for reproductive and maternal health (mean US$0.872 per capita in the Obama period, 2019) is associated with 25 additional maternal deaths per 100 000 live births (95% CI 7.94 to 41.18) under the GGR (table 3A, column 1). The magnitude of this effect suggests that the GGR erodes a meaningful share of the progress made in reducing maternal mortality over recent decades.

**Table 3:** Impact of the Global Gag Rule (GGR) on maternal mortality and reproductive health outcomes (1985–2023), continuous exposure measure

	Full sample	Continents		
		Africa	Latin America	Asia
**(A) Maternal mortality ratio (per 100 000 live births)**				
Aid per capita×GGR	24.562**	26.293	26.192***	18.452*
	(8.411)	(16.686)	(3.066)	(9.935)
	(7.943 to 41.182)	(−7.084 to 59.670)	(19.931 to 32.453)	(−1.442 to 38.346)
Observations	5850	2379	1209	2262
Countries	150	61	31	58
Mean of Dep. Var.	293.8	522.9	112.8	149.7
Effect relative to mean Dep. Var. (%)	8.4	5.0	23.2	12.3
**(B) Births attended by skilled health personnel (% of live births)**				
Aid per capita×GGR	−0.955**	0.247	−1.655**	−1.659**
	(0.432)	(0.839)	(0.739)	(0.595)
	(−1.808 to –0.102)	(−1.431 to 1.926)	(−3.164 to –0.147)	(−2.851 to –0.467)
Observations	2196	469	633	1089
Countries	150	61	31	58
Mean of Dep. Var.	85.7	65.7	92.2	90.6
Effect relative to mean Dep. Var. (%)	−1.1	0.4	−1.8	−1.8
**(C) Contraceptive prevalence, modern methods (% women aged 15–49 years)**				
Aid per capita×GGR	−0.663**	−0.917**	−0.335	−0.789**
	(0.310)	(0.446)	(0.868)	(0.373)
	(−1.276 to –0.050)	(−1.810 to –0.024)	(−2.127 to 1.457)	(−1.538 to –0.041)
Observations	923	419	159	341
Countries	137	60	25	52
Mean of Dep. Var.	36.0	25.4	52.4	41.2
Effect relative to mean Dep. Var. (%)	−1.8	−3.6	−0.6	−1.9
**(D) Unmet need for contraception (% of married women aged 15–49 years)**				
Aid per capita×GGR	−0.018	−0.745	–	2.743*
	(0.352)	(0.553)	–	(1.311)
	(−0.722 to 0.687)	(−1.868 to 0.379)	–	(−0.115 to 5.600)
Observations	226	139	–	45
Countries	57	36	–	13
Mean of Dep. Var.	11.7	9.1	–	10.6
Effect relative to mean Dep. Var. (%)	−0.2	−8.2	–	25.8

* p<0.10, ** p<0.05, *** p<0.01.

Each column reports coefficients from regressions of maternal health outcomes on a country’s reproductive and maternal health aid per capita during the Obama period (2009–2016) interacted with an indicator for whether the GGR is in force.

Panel A uses the maternal mortality ratio (per 100 000 live births) as the dependent variable. Panel B uses the share of births attended by skilled health personnel (% of live births). Panel C uses contraceptive prevalence, modern methods (% of women aged 15–49 years). Panel D uses unmet need for contraception (% of married women aged 15–49 years).

Estimates for unmet need for contraception are not reported for Latin America due to insufficient data for that outcome in the region. Every regression includes country and year fixed effects (not shown) to capture country-level heterogeneity and common trends.

Robust standard errors clustered at the country level in parentheses with 95% CIs.

Dep. Var., dependent variable.

The analysis of intermediate outcomes (tables2BD and 3B) shows that, in the binary exposure specification, the estimated effects on skilled birth attendance and contraceptive prevalence are negative although not statistically significant at conventional levels (table 2B,C). However, once we use the more general specification with a continuous aid per capita measure, these coefficients become negative and statistically significant in the full sample and in most regions (table 3B,C). Specifically, the GGR decreases the proportion of skilled birth attendance by 0.96 percentage points (95% CI −1.81 to −0.10), approximately a 1% reduction, and reduces contraceptive prevalence by 0.66 percentage points (95% CI −1.28 to −0.05), corresponding to roughly a 2% decrease.

For unmet need for contraception, the binary specification yields a marginally insignificant increase of 1.6 percentage points (95% CI −0.12 to 3.34) (table 2D), corresponding to a 13.7% rise relative to the mean of the dependent variable (mean=11.7 in highly exposed countries). Data on unmet need for contraception are available only for a subset of countries (n=57), and estimates are therefore not reported for Latin America due to insufficient data for this outcome in the region. In the binary exposure specification (table 2D), the estimates are insignificant for Africa whereas in Asia the GGR is associated with a sizeable and statistically significant increase in unmet need of about 3.5 percentage points (95% CI 1.13 to 5.81). In the continuous specification (table 3D), the average effect is close to zero but the results similarly suggest sizeable—though marginally insignificant—increases in unmet need for contraception in Asia.

Turning to the robustness analysis, our findings suggest that the results are robust to redefining baseline aid dependence using the Clinton administration period (1993–2000) rather than the Obama administration (online supplemental table A.1); the inclusion of time-varying covariates such as GDP per capita and secondary school enrolment (online supplemental table A.2); and the exclusion of the COVID-19 pandemic years 2020 and 2021 (online supplemental table A.3).

## Discussion

The recent expansion of the GGR under President Trump has transformed the landscape of US foreign health assistance. Rebranded as the Protecting Life in Global Health Assistance policy, it extended abortion-related funding restrictions to nearly all US global health programmes, thereby reshaping the organisation and funding of reproductive healthcare and family planning in many developing countries.^29^ In addition, significant changes to the structure and financing of the USAID have had far-reaching implications. In 2025 the administration implemented a 90-day freeze on all foreign assistance, followed by the dissolution of USAID. Although an Emergency Humanitarian Waiver to the Foreign Assistance Pause was issued in January 2025 by the Trump administration, the waiver excluded all activities involving abortion and family planning. An estimated 80% of USAID programmes were discontinued,^9^ with potentially large effects on health outcomes and human capital formation in low-income countries. According to the Organisation for Economic Co-operation and Development (OECD),^4^ cuts to ODA are not confined to the USA but are also evident in several European countries including the UK, France and Germany.

Our findings are relevant in light of these recent reductions in foreign aid, and in the context of the United Nations Sustainable Development Goals for health (Goal 3) and gender equality (Goal 5). They provide the first large-scale quantitative estimates of the maternal mortality consequences of the GGR, showing that US policy shifts can have measurable impacts on global health outcomes within the relatively short time frame of a Presidential administration. An estimated 11% increase in maternal deaths corresponds to roughly one-fifth of the global decline in maternal mortality achieved between 1985 and 2024. Maternal mortality represents only the visible portion of a much larger set of reproductive health risks including haemorrhage, sepsis, reproductive tract infections, uterine perforation, cervical tears, chronic pain, infertility and elevated risks in subsequent pregnancies.^21^ The findings highlight the sensitivity of global health outcomes to fluctuations in international aid priorities and the importance of policy continuity to sustain progress towards global health and gender equity objectives.

This study has several limitations. First, our empirical strategy may be subject to unobserved confounding if US Presidential election outcomes correlate with other global factors influencing maternal health, such as compensating aid flows from other countries. As any compensating flows are triggered by US aid cuts, our estimates capture the net impacts of US aid cuts. Second, the control group in our analysis is partially treated since countries with below median aid exposure still receive some US assistance. Thus, our estimates for the impact of the GGR-led cuts on maternal mortality are biased downwards and may be thought of as a lower bound. Third, while maternal mortality serves as a critical indicator, it captures only the most severe outcome and underlying morbidity—often unrecorded or mismeasured—remains difficult to quantify. An additional potential limitation of our analysis relates to the characteristics of aggregated maternal mortality data. Although the MMR series compiled in the World Development Indicators is widely used and draws on the best available country-level sources, maternal deaths may be under-reported or misclassified, particularly in low- and middle-income settings. In addition, because the estimates involve model-based adjustments to harmonise data of varying quality, short-term fluctuations in maternal health outcomes are smoothed out. These features do not preclude meaningful analysis but should be kept in mind when interpreting year-to-year variation. Finally, some intermediate outcomes—most notably unmet need for contraception—are available only for a subset of countries (57 in total), which narrows the coverage of the supplementary analyses.

Despite these potential limitations, this study contributes to the limited empirical evidence on the health effects of the GGR by providing large-scale quantitative estimates based on nearly four decades of data for approximately 150 countries. Importantly, it shows estimates for the three developing country regions, demonstrating impacts in Latin America and Asia and not just Africa. Using a quasi-experimental design to capture causal impacts, our analysis offers evidence of how international political shifts and aid conditionalities can shape maternal health and influence broader trajectories of global development. Our results cohere with a broader body of evidence showing that being denied an abortion can have both immediate and long-term consequences, including poorer physical health, reduced mental well-being and adverse socioeconomic outcomes.^30^,^32^

## Conclusion

Using data for four decades covering 150 countries and each of the developing country continents, we estimate the effects of US aid restrictions on maternal mortality. Our findings indicate that the GGR has had substantial consequences for maternal health worldwide. Countries more reliant on US family planning aid experienced significant increases in maternal mortality corresponding to a rise of 11% following reinstatement of the policy, offsetting a considerable share of the global progress achieved since 1985. Evidence on mechanisms suggests a decline in contraceptive use and reductions in skilled birth attendance. These results underscore the vulnerability of health systems to abrupt shifts in donor policy and highlight the importance of stable international support for reproductive health services. Although the policy named Protecting Life in Global Health Assistance under the Trump administration aims to protect life, our findings instead suggest higher rates of maternal deaths related to pregnancy and childbirth.

## Supplementary material

10.1136/bmjgh-2025-020223 online supplemental file 1

## Data Availability

Data are available in a public, open access repository.
