# Supplementary material for: US Presidential Party switches are mirrored in global maternal mortality
Source: BMJ Glob Health. 2026 Mar 24;11(3):e020223. doi: 10.1136/bmjgh-2025-020223 (PMC13157751; doi:10.1136/bmjgh-2025-020223)
Supplement: online supplemental file 1 [file bmjgh-11-3-s001.pdf]

Online Supplementary Material

"US Presidential Party Switches Are Mirrored in Global Maternal Mortality"

Sonia Bhalotra, PhD, University of Warwick, UK
Damian Clarke, PhD, Universidad de Chile, Chile, and University Exeter, UK
Manuel Fernandez, PhD, Universidad de los Andes, Colombia
Hanna Mühlrad, PhD, Karolinska Institutet, Sweden

A Figures and Tables

Figure A.1: Conceptual Model

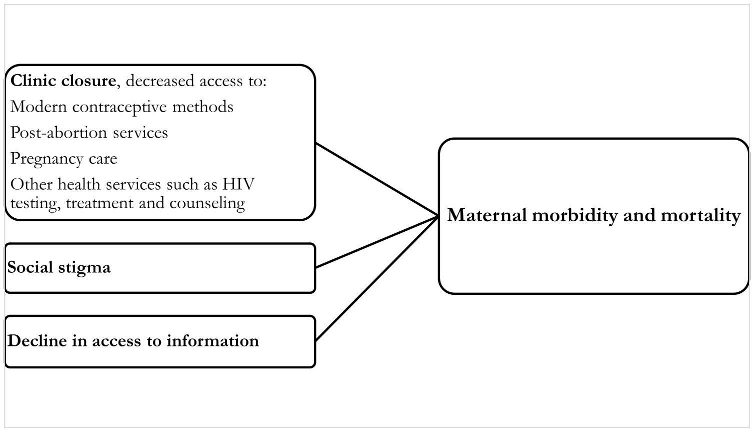

Notes: Simple conceptual model of the causal pathway

Figure A.2: Country Classification by Exposure to the Global Gag Rule (Obama-Era Aid Measure)

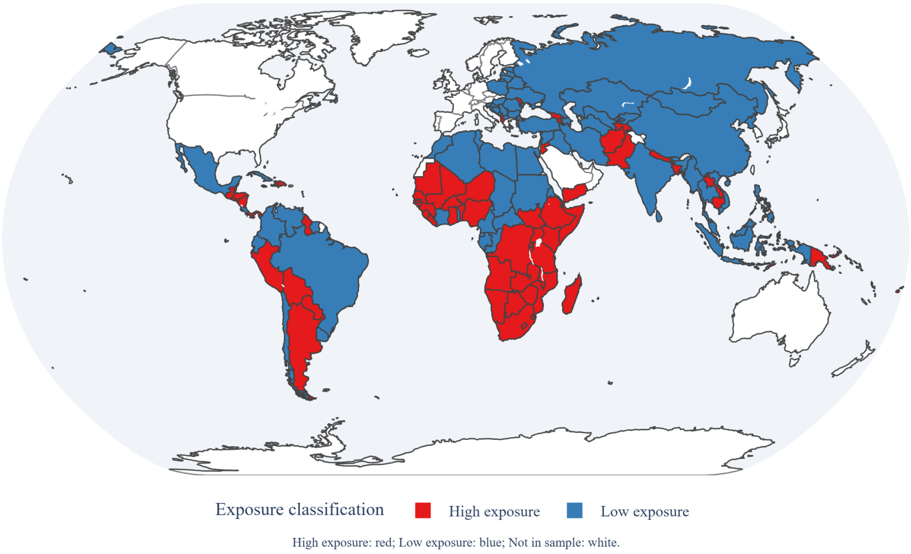

Notes: Countries in red are classified as high exposure and countries in blue as low exposure. Exposure is defined using average per-capita U.S. Official Development Assistance (ODA) for health targeted to reproductive and maternal health during the Obama administration (2009–2016), with high exposure indicating above-median aid. Countries shown in white are not included in the estimation sample because they receive no U.S. ODA for reproductive and maternal health.

Table A.1: Impact of the Global Gag Rule on Maternal Mortality and Reproductive Health Outcomes (1985-2024), Binary Exposure Based on Clinton-Era Aid Levels

		Continents		
	Full Sample	Africa	Lat. Am.	Asia
**Panel A: Maternal mortality ratio (per 100,000 live births)**				
High Exposure (Clinton) × GGR	31.988**	56.292**	17.435*	-4.407
	(12.637)	(21.795)	(10.043)	(16.095)
	[7.017,56.959]	[12.694,99.889]	[-3.076,37.946]	[-36.637,27.823]
Observations	5,850	2,379	1,209	2,262
Countries	150	61	31	58
Mean of Dep. Var.	427.7	626.9	154.4	269.8
Effect Relative to Mean Dep. Var. (%)	7.5	9.0	11.3	-1.6
**Panel B: Births attended by skilled health personnel (% of live births)**				
High Exposure (Clinton) × GGR	-1.124*	0.725	-2.012**	-0.728
	(0.600)	(1.662)	(0.951)	(0.944)
	[-2.310,0.062]	[-2.599,4.050]	[-3.955,-0.069]	[-2.618,1.163]
Observations	2,196	469	633	1,089
Countries	150	61	31	58
Mean of Dep. Var.	75.3	59.5	86.3	79.6
Effect Relative to Mean Dep. Var. (%)	-1.5	1.2	-2.3	-0.9
**Panel C: Contraceptive prevalence, modern methods (% women 15–49)**				
High Exposure (Clinton) × GGR	-1.556*	-2.102*	-4.193*	-0.548
	(0.836)	(1.204)	(2.146)	(1.169)
	[-3.210,0.097]	[-4.512,0.307]	[-8.622,0.236]	[-2.895,1.800]
Observations	923	419	159	341
Countries	137	60	25	52
Mean of Dep. Var.	29.7	23.8	47.9	28.1
Effect Relative to Mean Dep. Var. (%)	-5.2	-8.8	-8.7	-1.9
**Panel D: Unmet need for contraception (% of married women ages 15–49)**				
High Exposure (Clinton) × GGR	0.275	-1.409	-	3.431*
	(1.248)	(2.471)	-	(1.730)
	[-2.225,2.774]	[-6.425,3.608]	-	[-0.338,7.199]
Observations	226	139	-	45
Countries	57	36	-	13
Mean of Dep. Var.	11.7	9.4	-	8.9
Effect Relative to Mean Dep. Var. (%)	2.3	-15.0	-	38.5

Notes: Each column reports coefficients from regressions of maternal health outcomes on a country's degree of exposure to the GGR interacted with whether the GGR is enacted. High-exposure countries are defined as those with reproductive and maternal health aid per capita above the sample median during the Clinton period (1993–2000). Panel A uses the maternal mortality ratio (per 100,000 live births) as the dependent variable. Panel B uses the share of births attended by skilled health personnel (% of live births). Panel C uses contraceptive prevalence, modern methods (% of women aged 15–49). Panel D uses unmet need for contraception (% of married women ages 15–49). Estimates for unmet need for contraception are not reported for Latin America due to insufficient data for that outcome in the region. Every regression includes country and year fixed effects (not shown) to capture country-level heterogeneity and common trends. Robust standard errors clustered at the country level in parentheses; 95 percent confidence intervals are reported in square brackets.
* $p < 0.10$, ** $p < 0.05$, *** $p < 0.01$.

Table A.2: Impact of the Global Gag Rule on Maternal Mortality and Reproductive Health Outcomes (1985-2024), Binary Exposure and Controls for GDP and Female Gross Enrollment Ratio in Secondary (% of Relevant Age Group)

		Continents		
	Full Sample	Africa	Lat. Am.	Asia
**Panel A: Maternal mortality ratio (per 100,000 live births)**				
High Exposure × GGR	39.222***	55.485**	5.291	43.729**
	(10.868)	(21.051)	(10.967)	(13.881)
	[17.743,60.701]	[13.363,97.607]	[-17.138,27.720]	[15.923,71.536]
Observations	3,137	1,237	707	1,193
Countries	147	60	30	57
Mean of Dep. Var.	357.1	544.2	111.6	242.9
Effect Relative to Mean Dep. Var. (%)	11.0	10.2	4.7	18.0
**Panel B: Births attended by skilled health personnel (% of live births)**				
High Exposure × GGR	-1.304	0.024	-1.532	-1.271
	(0.882)	(2.289)	(1.100)	(1.419)
	[-3.048,0.441]	[-4.576,4.624]	[-3.782,0.717]	[-4.118,1.576]
Observations	1,354	277	434	635
Countries	134	50	30	54
Mean of Dep. Var.	76.6	62.9	88.9	74.2
Effect Relative to Mean Dep. Var. (%)	-1.7	0.0	-1.7	-1.7
**Panel C: Contraceptive prevalence, modern methods (% women 15–49)**				
High Exposure × GGR	-0.703	-0.330	-0.777	1.217
	(0.978)	(1.745)	(3.947)	(1.572)
	[-2.642,1.236]	[-3.842,3.182]	[-9.038,7.483]	[-1.962,4.395]
Observations	548	245	93	196
Countries	107	47	20	40
Mean of Dep. Var.	32.8	25.3	53.5	30.0
Effect Relative to Mean Dep. Var. (%)	-2.1	-1.3	-1.5	4.1
**Panel D: Unmet need for contraception (% of married women ages 15–49)**				
High Exposure × GGR	0.184	-2.131	-	0.081
	(1.666)	(4.979)	-	(3.377)
	[-3.180,3.548]	[-12.486,8.224]	-	[-7.706,7.868]
Observations	129	66	-	25
Countries	42	22	-	9
Mean of Dep. Var.	12.1	9.7	-	9.4
Effect Relative to Mean Dep. Var. (%)	1.5	-21.9	-	0.9

Notes: Each column reports coefficients from regressions of maternal health outcomes on a country's degree of exposure to the GGR interacted with whether the GGR is enacted. High-exposure countries are defined as those with reproductive and maternal health aid per capita above the sample median during the Obama period (2009–2016). Panel A uses the maternal mortality ratio (per 100,000 live births) as the dependent variable. Panel B uses the share of births attended by skilled health personnel (% of live births). Panel C uses contraceptive prevalence, modern methods (% of women aged 15–49). Panel D uses unmet need for contraception (% of married women ages 15–49). Estimates for unmet need for contraception are not reported for Latin America due to insufficient data for that outcome in the region. Every regression includes country and year fixed effects (not shown) to capture country-level heterogeneity and common trends. Estimates for wanted fertility are not reported for Latin America due to insufficient data for that outcome in the region. Robust standard errors clustered at the country level in parentheses; 95 percent confidence intervals are reported in square brackets.

* $p < 0.10$, ** $p < 0.05$, *** $p < 0.01$.

Table A.3: Impact of the Global Gag Rule on Maternal Mortality and Reproductive Health Outcomes (1985-2024), Binary Exposure; Excluding COVID-19 Years 2020–2021

		Continents		
	Full Sample	Africa	Lat. Am.	Asia
**Panel A: Maternal mortality ratio (per 100,000 live births)**				
High Exposure × GGR	47.224***	46.564*	26.723**	41.207**
	(12.946)	(24.368)	(9.887)	(20.291)
	[21.643,72.805]	[-2.179,95.308]	[6.530,46.915]	[0.575,81.838]
Observations	5,550	2,257	1,147	2,146
Countries	150	61	31	58
Mean of Dep. Var.	437.7	642.4	155.8	276.4
Effect Relative to Mean Dep. Var. (%)	10.8	7.2	17.2	14.9
**Panel B: Births attended by skilled health personnel (% of live births)**				
High Exposure × GGR	-1.079*	-0.663	-1.003	-1.086
	(0.630)	(1.741)	(0.915)	(1.111)
	[-2.324,0.166]	[-4.145,2.820]	[-2.871,0.865]	[-3.311,1.139]
Observations	2,139	453	623	1,059
Countries	150	61	31	58
Mean of Dep. Var.	75.0	59.0	86.2	79.2
Effect Relative to Mean Dep. Var. (%)	-1.4	-1.1	-1.2	-1.4
**Panel C: Contraceptive prevalence, modern methods (% women 15–49)**				
High Exposure × GGR	-1.214	-1.482	-2.257	-1.419
	(0.816)	(1.225)	(2.041)	(1.460)
	[-2.829,0.401]	[-3.933,0.970]	[-6.478,1.965]	[-4.352,1.514]
Observations	887	399	153	331
Countries	134	60	24	50
Mean of Dep. Var.	29.1	22.9	47.5	28.2
Effect Relative to Mean Dep. Var. (%)	-4.2	-6.5	-4.8	-5.0
**Panel D: Unmet need for contraception (% of married women ages 15–49)**				
High Exposure × GGR	1.773*	0.620	-	3.469**
	(0.918)	(1.845)	-	(1.075)
	[-0.067,3.614]	[-3.134,4.373]	-	[1.128,5.811]
Observations	217	131	-	45
Countries	55	34	-	13
Mean of Dep. Var.	11.9	9.5	-	8.9
Effect Relative to Mean Dep. Var. (%)	14.9	6.5	-	39.0

Notes: Each column reports coefficients from regressions of maternal health outcomes on a country's degree of exposure to the GGR interacted with whether the GGR is enacted. High-exposure countries are defined as those with reproductive and maternal health aid per capita above the sample median during the Obama period (2009–2016). Panel A uses the maternal mortality ratio (per 100,000 live births) as the dependent variable. Panel B uses the share of births attended by skilled health personnel (% of live births). Panel C uses contraceptive prevalence, modern methods (% of women aged 15–49). Panel D uses unmet need for contraception (% of married women ages 15–49). Estimates for unmet need for contraception are not reported for Latin America due to insufficient data for that outcome in the region. Every regression includes country and year fixed effects (not shown) to capture country-level heterogeneity and common trends. Robust standard errors clustered at the country level in parentheses; 95 percent confidence intervals are reported in square brackets..

* $p < 0.10$, ** $p < 0.05$, *** $p < 0.01$.

Table A.4: Data Availability by Country, Exposure Classification, and Number of Non-Missing Observations by Outcome

	Exposure Classification		Obs. per Country			
	High Exposure (Obama)	High Exposure (Clinton)	Maternal Mortality Ratio	Births Attended Skilled	Contraceptive Prevalence (Modern)	Unmet Need for Contraception
Afghanistan	1	0	39	12	9	1
Angola	1	1	39	4	4	1
Albania	1	1	39	20	5	2
Argentina	1	1	39	27	2	0
Armenia	1	1	39	27	6	4
Antigua and Barbuda	0	0	39	20	0	0
Azerbaijan	0	1	39	33	4	1
Burundi	1	1	39	11	9	2
Benin	1	1	39	7	7	4
Burkina Faso	1	1	39	6	13	4
Bangladesh	1	1	39	17	17	5
Bulgaria	0	0	39	31	4	0
Bosnia and Herzegovina	0	0	39	26	3	0
Belarus	0	0	39	29	5	0
Belize	1	1	39	23	4	0
Bolivia	1	1	39	17	7	3
Brazil	0	0	39	26	4	1
Bhutan	0	0	39	20	2	0
Botswana	1	1	39	20	4	0
Central African Republic	0	1	39	7	5	0
Chile	0	0	39	27	2	0
China	0	0	39	26	9	0
Cote d'Ivoire	0	1	39	9	10	2
Cameroon	0	0	39	8	7	4
Congo, Dem. Rep.	1	0	39	5	7	2
Congo, Rep.	0	0	39	3	3	2
Colombia	0	0	39	25	7	6
Comoros	1	1	39	3	4	1
Cabo Verde	0	0	39	15	4	0
Costa Rica	0	0	39	25	6	0
Cuba	0	0	39	30	6	0
Djibouti	1	1	39	3	3	0
Dominica	0	0	39	23	1	0
Dominican Republic	1	1	39	18	12	6
Algeria	0	0	39	6	8	0
Ecuador	0	1	39	24	8	0
Egypt, Arab Rep.	0	1	39	14	12	7
Eritrea	0	1	39	4	3	1
Estonia	0	0	39	30	5	0
Ethiopia	1	0	39	9	13	4
Fiji	1	0	39	27	1	0
Micronesia, Fed. Sts.	1	1	39	10	0	0
Gabon	0	0	39	3	3	3
Georgia	1	1	39	29	6	0
Ghana	1	1	39	13	14	4
Guinea	1	1	39	9	6	4
Gambia, The	0	1	39	7	7	2
Guinea-Bissau	1	1	39	6	5	0
Equatorial Guinea	0	0	39	3	2	0
Grenada	0	0	39	16	1	0
Guatemala	1	1	39	16	6	3
Guyana	1	0	39	21	7	1
Honduras	1	1	39	10	8	2
Croatia	0	0	39	30	0	0
Haiti	1	1	39	8	7	5
Hungary	0	0	39	29	3	0
Indonesia	0	0	39	21	31	7

continued on next page

Table A.4 – *continued from previous page*

	Exposure Classification		Obs. per Country			
	High Exposure (Obama)	High Exposure (Clinton)	Maternal Mortality Ratio	Births Attended Skilled	Contraceptive Prevalence (Modern)	Unmet Need for Contraception
India	0	0	39	8	9	3
Iran, Islamic Rep.	0	0	39	9	10	0
Iraq	0	0	39	7	5	0
Jamaica	1	1	39	22	6	0
Jordan	1	1	39	6	8	7
Kazakhstan	0	0	39	30	6	1
Kenya	1	1	39	8	15	4
Kyrgyz Republic	0	1	39	28	5	1
Cambodia	1	1	39	11	7	4
Kiribati	1	1	39	10	2	0
St. Kitts and Nevis	1	0	39	19	0	0
Lao PDR	1	0	39	7	5	0
Lebanon	0	0	39	3	5	0
Liberia	1	1	39	5	4	3
Libya	0	0	39	8	4	0
St. Lucia	0	1	39	17	1	0
Sri Lanka	0	0	39	6	5	0
Lesotho	1	0	39	8	9	3
Lithuania	0	0	39	33	3	0
Latvia	0	0	39	29	1	0
Morocco	0	1	39	9	8	2
Moldova	1	1	39	33	5	1
Madagascar	1	1	39	8	9	4
Maldives	0	0	39	14	5	2
Mexico	0	0	39	21	11	0
Marshall Islands	1	1	39	7	1	0
North Macedonia	0	0	39	33	2	0
Mali	1	1	39	8	8	4
Myanmar	0	0	39	7	5	1
Montenegro	0	1	39	12	4	0
Mongolia	0	1	39	10	9	0
Mozambique	1	1	39	5	7	3
Mauritania	1	1	39	6	6	2
Mauritius	0	0	39	27	4	0
Malawi	1	1	39	11	10	5
Malaysia	0	0	39	23	6	0
Namibia	1	1	39	4	5	4
Niger	1	1	39	8	11	3
Nigeria	1	0	39	9	12	5
Nicaragua	1	1	39	18	5	2
Nepal	1	1	39	11	12	4
Pakistan	1	1	39	17	12	4
Panama	1	0	39	28	4	0
Peru	1	1	39	20	21	9
Philippines	0	1	39	9	18	6
Palau	1	0	39	20	0	0
Papua New Guinea	1	1	39	7	3	1
Poland	0	0	39	34	3	0
Korea, Dem. People's Rep.	0	0	39	4	8	0
Paraguay	1	1	39	24	7	1
Palestine	1	1	39	8	6	0
Romania	0	0	39	32	4	0
Russian Federation	0	0	39	32	8	0
Rwanda	1	1	39	8	7	7
Sudan	0	0	39	4	6	0
Senegal	1	1	39	16	14	11
Solomon Islands	1	1	39	5	2	0
Sierra Leone	1	1	39	7	7	3
El Salvador	1	1	39	19	8	0
Somalia	1	0	39	5	3	0

continued on next page

Table A.4 – *continued from previous page*

	Exposure Classification		Obs. per Country			
	High Exposure (Obama)	High Exposure (Clinton)	Maternal Mortality Ratio	Births Attended Skilled	Contraceptive Prevalence (Modern)	Unmet Need for Contraception
Serbia	0	0	39	18	5	0
South Sudan	1	1	39	3	2	0
Sao Tome and Principe	1	0	39	6	5	1
Suriname	0	0	39	11	4	0
Slovenia	0	0	39	24	1	0
Eswatini	1	1	39	6	6	1
Seychelles	0	0	39	13	0	0
Syrian Arab Republic	0	0	39	4	4	0
Chad	0	1	39	6	6	3
Togo	0	0	39	8	7	1
Thailand	0	0	39	8	12	0
Tajikistan	1	0	39	25	5	2
Turkmenistan	0	0	39	9	4	1
Timor-Leste	1	0	39	5	8	2
Tonga	1	1	39	16	2	0
Trinidad and Tobago	0	0	39	22	5	0
Tunisia	0	0	39	7	8	0
Turkey	0	0	39	13	7	5
Tuvalu	1	1	39	6	2	0
Tanzania	1	1	39	8	9	6
Uganda	1	1	39	7	14	4
Ukraine	0	0	39	26	4	1
Uruguay	0	1	39	31	2	0
Uzbekistan	0	1	39	26	5	0
St. Vincent and the Grenadines	0	0	39	18	0	0
Venezuela, RB	0	0	39	11	2	0
Vietnam	0	0	39	7	23	0
Vanuatu	0	1	39	10	3	0
Samoa	1	0	39	7	3	0
Kosovo	0	0	0	1	2	0
Yemen, Rep.	1	1	39	6	5	2
South Africa	1	0	39	7	4	2
Zambia	1	1	39	9	6	6
Zimbabwe	1	1	39	9	9	4
